# Silica–phenolic hybrid nanocarriers in redox oncology: interfacial mechanisms and translational barriers

**DOI:** 10.3389/fbioe.2026.1814492

**Published:** 2026-05-04

**Authors:** José Roberto Vega-Baudrit, Yendry Regina Corrales-Ureña

**Affiliations:** 1 National Nanotechnology Laboratory LANOTEC CENAT CONARE, San José, Costa Rica; 2 Chemistry School, Universidad Nacional, Heredia, Costa Rica

**Keywords:** controlled release, polyphenols, protein corona, mesoporous silica nanoparticles, tumor microenvironment

## Abstract

Plant-derived phenolic compounds are attractive redox-active agents in oncology because they can reshape oxidative stress and redox signaling; however, clinical translation remains limited by low solubility, instability, rapid metabolism, and insufficient tumor exposure. Mesoporous silica nanoparticles (MSNs) offer a modular scaffold that can stabilize phenolics, tune spatiotemporal release, and program interfacial redox kinetics. In this mini-review, we argue that silica-phenolic systems are better understood as programmable redox interfaces whose outputs depend on dose, compartment, and microenvironment. We define tumor redox sensitization as tumor-directed amplification of oxidative pressure and antioxidant shielding as restricted protection of non-malignant tissues from therapy-associated oxidative injury. We discuss adsorption, covalent display, and stimuli-responsive gating, together with liabilities such as polyphenol pro-oxidant switching, protein corona-driven identity shifts, heterogeneous tumor exposure, safety and biodegradation constraints, and the absence of standardized redox readouts. We also place MSN-phenolic designs in the context of human evidence from adjacent silica nanomedicines and nanoformulated polyphenols, underscoring that oncology-specific MSN-phenolic systems remain preclinical. We propose practical design and reporting rules to improve reproducibility and clinical readiness.

## 1 Introduction

Redox dysregulation is a defining feature of malignant transformation and progression. Many tumors operate under elevated basal reactive oxygen species (ROS) generated by oncogenic signaling, mitochondrial dysfunction, and metabolic rewiring; moderate ROS can sustain pro-survival pathways, whereas excessive ROS can exceed antioxidant capacity and trigger cell death (Reczek and Chandel, 2017; Trachootham et al., 2009). This ‘tipping-point’ behavior makes redox biology an appealing engineering target—but it also demands precise, compartment-specific control. Recent work synthesizes actionable strategies and paradoxes in ROS targeting, reinforcing the rationale for spatially controlled redox modulation (Glorieux et al., 2024).

Plant-derived polyphenols (phenolic acids, flavonoids, stilbenes, and related metabolites) (Scalbert et al., 2005; Panche et al., 2016) can influence both oxidative stress and redox signaling through hydrogen-atom transfer and single-electron transfer chemistry, metal chelation, and pathway modulation (Nuclear Factor kappa B ( NF-κB) / Nuclear factor erythroid 2–related factor 2 (Nrf2)). However, robust *in vitro* effects often fail to translate into clinical effects because many phenolics exhibit poor solubility, instability, and rapid metabolism, which lead to low systemic bioavailability (e.g low levels in the bloodstream) and inadequate tumor exposure (Manach et al., 2004; Anand et al., 2007; D’Archivio et al., 2010).

In this review, redox sensitization refers to a tumor-directed increase in oxidative pressure, or a reduction in tumor antioxidant buffering, sufficient to move malignant cells closer to an apoptotic or therapy-responsive threshold. By contrast, antioxidant shielding refers to a deliberately restricted protection of non-malignant tissues from therapy-associated oxidative damage without restoring redox fitness in tumor cells. We introduce these definitions early because the same phenolic cargo can behave as an antioxidant, a pro-oxidant, or a signaling modulator depending on dose, local metal availability, pH, compartmentalization, and the surrounding biomolecular milieu (Galati and O'Brien, 2004; León-González et al., 2015; Vrankova et al., 2026).

Mesoporous silica nanoparticles (MSNs) provide a modular platform to partially decouple intrinsic phenolic chemistry from *in vivo* performance. Their value lies not only in high loading capacity, but also in the ability to program interfacial redox kinetics using pore architecture and surface chemistry (Vallet-Regí et al., 2001; Slowing et al., 2008; Tang et al., 2012). We therefore frame silica-phenolic hybrids as programmable redox interfaces rather than generic antioxidant carriers. This mini-review (i) maps the interfacial design space that governs phenolic stabilization and release, (ii) formalizes a dual redox behavior model for tumors and healthy tissues, (iii) situates silica platforms within the broader nanocarrier landscape, and (iv) identifies translational gates and reporting priorities that determine clinical readiness. In response to reviewer concerns, we also make the toxicity implications of dual redox behavior explicit and anchor the discussion against the still-limited human evidence surrounding silica nanomedicine and nanoformulated polyphenols.

## 2 Mesoporous silica nanoparticles as a redox engineering scaffold

### 2.1 Architecture and surface reactivity

MSNs are typically amorphous silica frameworks with ordered mesopores (commonly 2–10 nm in diameter for drug delivery) whose pore diameter, pore connectivity, particle size, and surface charge can be tuned through synthesis and post-functionalization (Tang et al., 2012; Mamaeva et al., 2013). These variables directly shape (i) phenolic loading capacity, (ii) diffusion and release kinetics, and (iii) colloidal stability and uptake. At physiological pH, silica surfaces are generally negatively charged due to deprotonated silanols, which influences electrostatic adsorption, corona formation, and endosomal trafficking (He and Shi, 2011). Recent cancer-focused reviews further highlight the integration of MSN architectures into multimodal theranostics and combination regimens (Dutta Gupta et al., 2024).

Silanol chemistry also underpins functionalization versatility. The population and heterogeneity of surface ≡Si–OH groups determine hydrogen-bonding propensity, hydrophilicity, and the density of anchor points for organosilane grafting (Zhuravlev, 2000). Practically, surface chemistry is not an aesthetic detail: it controls the biological identity of the particle upon exposure to serum proteins and therefore governs the gap between *in vitro* proof of concept and *in vivo* performance.

### 2.2 Biodegradation and safety as design constraints

Silica platforms are often described as biocompatible, but biosafety depends strongly on dose, particle size, porosity, surface functional groups, degradation rate, and residual contaminants (Croissant et al., 2018; Huang et al., 2022; Janjua et al., 2023). Hydrolytic dissolution to orthosilicic acid can support clearance, yet biodistribution frequently favors reticuloendothelial organs such as liver and spleen, especially for systems lacking stealth coatings. For redox-active cargos, this matters twice: the carrier can accumulate outside the tumor, and the payload can perturb oxidative balance in the same off-target compartments. Accordingly, biodegradation, inflammatory liability, and residual template control must be treated as front-end design variables rather than afterthoughts.

A second safety layer arises from the fact that phenolics are not uniformly protective. Depending on concentration, transition-metal availability, oxygen tension, and enzymatic context, polyphenols can generate semiquinones, quinones, hydrogen peroxide, or glutathione-consuming intermediates that shift a system from cytoprotection toward oxidative damage (Galati and O'Brien, 2004; León-González et al., 2015; Vrankova et al., 2026). An MSN formulation that improves intracellular retention may therefore increase therapeutic leverage in tumors, but it can also amplify pro-oxidant exposure in macrophages, hepatocytes, endothelial cells, or cardiomyocytes if biodistribution is poorly controlled. The translational question is not whether pro-oxidant behavior exists, but whether it is spatially confined and mechanistically justified.

## 3 Interfacial mechanisms of phenolic functionalization on MSNs

### 3.1 Adsorption and confinement-dominant loading

Adsorption-dominant loading is based mainly on non-covalent affinity between phenolics and the pore/surface environment. Hydrogen bonding, electrostatics (pH- and ionic-strength-dependent), and hydrophobic/π interactions collectively govern loading and desorption. Mesoporous confinement can stabilize labile phenolics by restricting mobility and limiting access to pro-oxidant species, thereby shifting behavior from rapid loss of activity to sustained redox availability.

### 3.2 Covalent display and stimuli-responsive gating

Covalent strategies tether phenolics (or phenolic-bearing linkers) to silica through organosilane chemistry, reducing premature leaching but potentially altering accessibility of redox-active motifs. Stimuli-responsive gating adds dynamic control at pore openings (e.g., polymer brushes or supramolecular caps), enabling conditional release driven by tumor-relevant cues such as acidic pH, elevated ROS, or intracellular reducing potential. Importantly, gating should be evaluated under serum-containing conditions because the corona can mask ligands and alter gate behavior.

### 3.3 From interface to intracellular redox availability

The core mechanistic question is not simply whether phenolics can be loaded, but whether interfacial design yields the intended intracellular redox kinetics (in terms of timing, dose, and compartment). Endosomal trafficking, lysosomal acidity, and local redox buffering can either enable controlled release or trap cargo. Accordingly, studies should report the identity of the materials (size distribution, porosity, surface chemistry), payload accounting (loading and stability), and post-serum identity before making strong claims about redox modulation.

**FIGURE 1:**
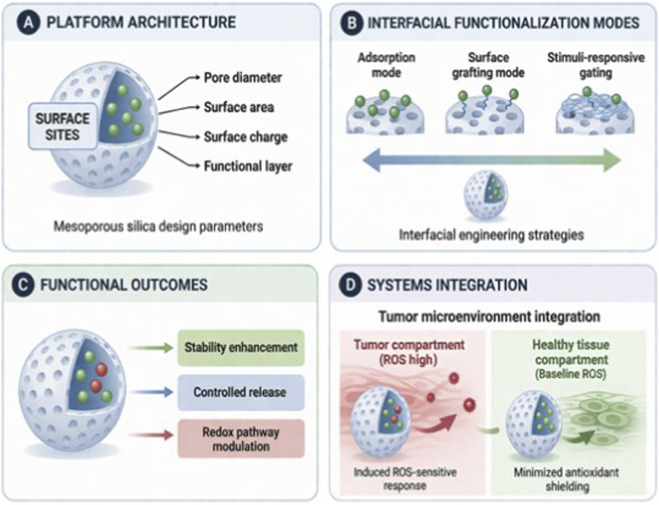
Interfacial design map for MSN-phenolic systems (conceptual; no molecular structures). The framework links **(A)** platform architecture and surface properties, **(B)** functionalization modes (adsorption, covalent display, stimuli-responsive gating), **(C)** functional outcomes (stability enhancement, controlled release, redox modulation), and **(D)** compartment-specific behavior in tumor versus healthy tissues.

Figure 1 summarizes a design map that links the MSN architecture and interfacial strategies to functional redox outcomes.

## 4 Dual redox behavior in oncology: tumor sensitization versus tissue protection

### 4.1 Tumor-localized redox sensitization

In tumors with high basal ROS, additional oxidative stress can push cells beyond tolerable limits and trigger apoptosis; conversely, indiscriminate antioxidant buffering can inadvertently support tumor survival by restoring redox homeostasis. This creates a design challenge: redox modulation must be targeted in space and time to avoid global suppression of ROS signaling, which may reduce therapeutic efficacy (Trachootham et al., 2009). Polyphenols further complicate the landscape because their activity can be concentration- and context-dependent, including pro-oxidant behavior under specific conditions. MSNs can help operationalize tumor-localized redox sensitization by controlling exposure kinetics and (when validated) coupling release to tumor cues.

### 4.2 Healthy-tissue antioxidant shielding and the antioxidant paradox

Many standard therapies cause systemic oxidative injury (e.g., cardiotoxicity from anthracyclines), prompting antioxidant shielding strategies (Conklin, 2004). However, clinical and pre-clinical debates on antioxidant supplementation in oncology underscore the paradox: if antioxidants protect tumor cells, they may attenuate ROS-dependent therapeutic mechanisms. Silica–phenolic systems are therefore only credible as ‘dual redox’ technologies when they plausibly enforce compartment-specific behavior—through differential accumulation, triggerable release, or logic-gated activation—rather than increasing systemic antioxidant exposure.

### 4.3 Safety implications of dual redox programming

The same formulation feature that is attractive for tumor sensitization, namely, prolonged intracellular residence or stimulus-linked release, can magnify liability when exposure occurs in non-target tissues. Polyphenols can undergo auto-oxidation, redox cycling, and quinone formation; these events may deplete GSH, covalently modify proteins, perturb mitochondrial function, and trigger inflammatory signaling if they occur in healthy compartments (Galati and O'Brien, 2004; León-González et al., 2015; Vrankova et al., 2026). For this reason, dual redox programming should never be evaluated solely by improved tumor killing. It must also be evaluated as a risk-management problem in which the acceptable therapeutic window depends on exposure geography.

Practically, this means that toxicity packages for MSN-phenolic systems should include matched non-malignant controls, hemocompatibility, macrophage and endothelial stress readouts, organ-level biodistribution, and time-resolved oxidative injury markers rather than relying only on acute viability assays. Claims that a system is protective in healthy tissue should be supported by direct evidence of reduced oxidative damage or preserved organ function under a relevant therapy challenge. Otherwise, antioxidant shielding remains an aspiration rather than a demonstrated property.

### 4.4 Representative evidence base: what has been shown and what remains uncertain

Representative studies report improved intracellular delivery and enhanced anticancer effects of MSN-loaded phenolics compared with free compounds, including curcumin-loaded MSNs (Kotcherlakota et al., 2016) and photodynamic therapy designs using curcumin-loaded MSNs (Kuang et al., 2020). Resveratrol-loaded, surface-modified MSNs have been reported to enhance antiproliferative effects in prostate cancer models (Chaudhary et al., 2019). Folate-targeted quercetin MSNs improved uptake in folate receptor-positive models (Sarkar et al., 2016; Mishra et al., 2020), and dual-delivery approaches (for example, phenolic plus siRNA) have been proposed to address resistance pathways (Song et al., 2020). These representative systems are summarized in Table 1 to improve manuscript usability and to make the translational signal of each study explicit.

**TABLE 1:** Representative MSN-phenolic systems relevant to redox oncology and their main translational message.

Representative system	MSN/Interfacial strategy	Experimental context and main outcome	Translational interpretation
Curcumin-loaded MSN (Kotcherlakota et al., 2016)	Adsorption and mesoporous confinement	Improved intracellular delivery and anticancer activity relative to free curcumin in preclinical cancer models	Supports the value of stabilization and controlled exposure, but serum identity and orthogonal redox readouts remain limited
PEGylated curcumin-loaded MSN for PDT (Kuang et al., 2020)	Curcumin loading plus PEG surface modification	Enhanced photodynamic therapy-associated killing in tumor models	Illustrates combination potential, yet biodistribution and compartment-specific redox kinetics require stronger validation
Resveratrol-loaded functionalized MSN (Chaudhary et al., 2019)	Surface-functionalized controlled release platform	Enhanced antiproliferative effects in prostate cancer models	Suggests better phenolic stabilization and uptake, but does not by itself resolve delivery ceiling or safety questions
Folate-targeted quercetin MSN (Sarkar et al., 2016)	Targeted loading with folate-directed uptake	Higher uptake and cytotoxicity in folate receptor-positive breast cancer cells	Targeting signal is promising, but must be revalidated after protein-corona formation and *in vivo* exposure heterogeneity
Magnetic folic-acid quercetin MSN (Mishra et al., 2020)	Multifunctional theranostic carrier	Quercetin delivery combined with imaging-oriented magnetic functionality	Theranostic complexity may add utility, but also increases chemistry, manufacturing, and control burden
Myricetin plus MRP-1 siRNA FA-conjugated MSN (Song et al., 2020)	Phenolic/nucleic-acid co-delivery	Improved suppressive effects in NSCLC models relative to phenolic monotherapy	Potentially useful for resistance modulation, but translational complexity and safety testing requirements increase substantially

**FIGURE 2:**
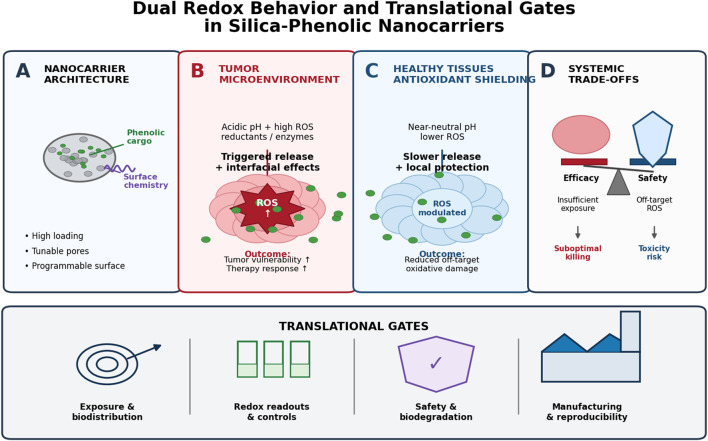
Conceptual framework of dual redox behavior in mesoporous silica nanoparticle (MSN)-phenolic systems. **(A)** MSN architecture enables high loading and programmable release through pore and surface engineering. **(B)** In the tumor microenvironment, acidity, elevated ROS, and reductants promote the release of phenolics and interfacial redox activity, leading to redox sensitization and enhanced therapeutic response. **(C)** In healthy tissues, near-neutral pH and lower ROS favor slower release and antioxidant shielding, reducing off-target oxidative damage. **(D)** Systemic outcomes depend on the balance between efficacy and safety; inadequate control can result in either suboptimal tumor killing or toxicity. Successful translation requires passing key gates: exposure and biodistribution; robust redox evidence with appropriate controls; safety and biodegradation; and manufacturability with batch-to-batch reproducibility.

However, many reports remain vulnerable to predictable reviewer critiques: redox endpoints are often single-probe or non-kinetic, post-serum identity is rarely assessed, and *in vivo* evidence for compartment-specific redox modulation is limited. The key message from the available literature is therefore not that dual redox behavior has been fully solved, but that MSNs offer a credible engineering route whose biological claims still require more rigorous mechanistic validation. Figure 2 summarizes the dual-compartment model and the translational gates that determine whether ‘dual redox behavior’ is achievable beyond *in vitro* settings.

## 5 Comparative perspective across nanocarrier classes

A comparative platform lens is included because translational feasibility is strongly platform-dependent, and interfacial redox programmability must be evaluated against manufacturing maturity, safety profiles, and regulatory precedent. MSNs should be selected based on engineering problems, not on platform preference. Polymeric nanoparticles (e.g., PLGA) can improve solubility and exposure with strong regulatory precedent, but degradation-driven release may be less programmable for multi-trigger redox logic (Danhier et al., 2012). Liposomes offer unmatched clinical maturity, yet membrane leakage and oxidative instability can be liabilities in ROS-rich contexts (Allen and Cullis, 2013). Chitosan systems offer biodegradability and cationic uptake advantages, but batch-to-batch variability and pH sensitivity can compromise reproducibility (Rinaudo, 2006). MOFs offer extreme loading and tunable pore environments, but metal-related safety and regulatory immaturity remain substantial barriers (Horcajada et al., 2012).

A pragmatic decision rule is as follows: choose MSNs when the primary requirement is architectural control over interfacial redox kinetics (stability + conditional release) and when a safety/reproducibility package can be credibly assembled; choose soft carriers when clinical maturity and manufacturing scalability dominate; and treat high-loading porous crystalline platforms as exploratory unless safety and stability are demonstrated early.

## 6 Translational barriers and clinical barriers

### 6.1 Biological identity shifts: protein corona and loss of targeting

A recurrent failure mode in nano-enabled targeting is the mismatch between ‘as-synthesized’ surfaces and the *in vivo* biological identity created by protein adsorption. Biomolecular coronas can redefine surface properties and mask targeting ligands, altering uptake pathways and biodistribution (Monopoli et al., 2012). In a canonical example, transferring-functionalized nanoparticles lost targeting capability after corona formation (Salvati et al., 2013). Consequently, claims of selective targeting or tumor specificity should be supported by post-serum characterization and, ideally, *in vivo* evidence. Methodologically, recent work shows that both corona isolation workflows and *in situ* characterization tools can materially change inferred corona fingerprints and downstream targeting conclusions (Bashiri et al., 2023; Fu et al., 2024; Sun et al., 2024) and highlight tissue-level consequences for targeted delivery (Chou and Lin, 2024).

### 6.2 Biodistribution heterogeneity and the delivery ceiling

Even with optimized surfaces, systemic nanoparticle delivery to solid tumors is often low and heterogeneous. A landmark analysis emphasized that only a small fraction of the injected dose reaches tumors across many preclinical systems (Wilhelm et al., 2016). Complementary mechanistic work has challenged simplistic gap-extravasation narratives and highlighted active trans-endothelial processes for nanoparticle entry in several models (Sindhwani et al., 2020). For dual redox designs, this exposure reality matters: partial tumor delivery may be insufficient for sensitization, while off-target exposure may still cause unwanted antioxidant shielding. Translation, therefore, requires exposure-aware design, quantifying benefit per delivered dose and validating the mechanism under realistic biodistribution constraints. Recent mechanistic reviews emphasize that tumor entry can be dominated by active transcytosis and other transvascular routes, with substantial heterogeneity across vessel states, arguing for biomarker-guided stratification rather than generic enhanced permeability and retention assumptions (Chan, 2023; Li et al., 2024).

### 6.3 Safety, biodegradation, and manufacturing reproducibility

Silica safety is context-dependent. Particle size, porosity, surface functional groups, and residual surfactant or solvent contaminants can drive inflammatory responses and organ accumulation (Croissant et al., 2018; Huang et al., 2022; Janjua et al., 2023). Manufacturing scale-up introduces additional risk because small variations in synthesis conditions can shift pore structure and functionalization density, altering release behavior, serum identity, and effective redox dose. When the active cargo itself can switch between antioxidant and pro-oxidant behavior, batch drift becomes mechanistically consequential rather than a purely analytical concern. Therefore, reproducibility metrics (batch-to-batch size, porosity, chemistry, and loading), residual controls, and storage-stability data should be treated as critical quality attributes.

### 6.4 Redox readout standardization: avoiding non-interpretable endpoints

Redox claims are frequently underspecified. Single-probe ROS dyes can be prone to interference, and endpoint-only assays rarely distinguish between radical scavenging and signaling modulation. For robust peer review, studies should combine at least two orthogonal redox readouts (e.g., ROS kinetics plus GSH/GSSG or lipid peroxidation), report time courses, and relate redox changes to mechanistic endpoints (e.g., death mode, pathway markers, or resistance phenotypes). Where feasible, authors should align ROS and oxidative damage assays with consensus best-practice guidance (Murphy et al., 2022) and report probe limitations, calibration, and orthogonal validation to keep redox claims interpretable.

### 6.5 Design rules and reporting considerations

Rigorous silica–phenolic development benefits from a minimal ‘evidence stack’ that connects materials identity to redox biology and translation. At a minimum, the platform definition should specify the silica type, the intended route, and the design rationale. Materials identity should include particle size distribution (transmission electron microscopy (TEM)/ dinamic light scattering (DLS)), porosity metrics (N_2_ sorption), confirmation of surface chemistry (e.g., Fourier Transform Infrared (FTIR) spectroscopy and X-ray photoelectron spectroscopy (XPS)), quantification of the organic fraction or grafting density (e.g., thermogravimetric analysis (TGA)), and evidence of template removal. Payload accounting should report loading method (adsorption/grafting/gating), loading efficiency, and phenolic stability during processing and storage. Release must be measured under serum-containing, biorelevant conditions with explicit trigger-response curves when gating is claimed. Post-serum identity (changes in size and ζ-potential; optional corona profiling) should be included for targeted systems. Finally, biological studies should distinguish redox buffering from redox sensitization using orthogonal redox readouts and appropriate controls (a free phenolic compound, a blank MSN, and a functionalized MSN without the payload). Supplementary Table S1 provides a condensed checklist designed for replication and peer-review clarity.

### 6.6 Current clinical evidence and adjacent trial landscape

The reviewer appropriately requested a clearer clinical perspective. At present, we are not aware of direct clinical trials of oncology-specific MSN-phenolic hybrids. This absence is important and should be stated plainly. What does exist is adjacent human evidence showing that silica nanoparticles can reach the clinic in other configurations. Ultrasmall silica C dots have demonstrated feasibility, renal clearance, and favorable safety in first-in-human oncologic imaging studies (Phillips et al., 2014), and ultrasmall core-shell fluorescent silica nanoparticles have been shown to be feasible and safe for image-guided sentinel lymph node biopsy in head and neck melanoma (Zanoni et al., 2021). Perspective and review articles now regard these programs as proof that silica nanoplatforms can be clinically translated, while also noting that mesoporous silica drug-delivery variants remain much less mature clinically (Janjua et al., 2021; Janjua et al., 2023).

The phenolic side of the interface also has a limited but emerging clinical footprint, as evidenced by adjacent nanomedicine research. Liposomal curcumin has reached phase I evaluation in patients with locally advanced or metastatic cancer (Greil et al., 2018), and intrapleural liposomal curcumin is currently under phase I clinical evaluation for malignant pleural effusion, with protocol-level evidence supporting its feasibility (Hocking et al., 2021). In addition, ClinicalTrials.gov lists a study of liposomal curcumin combined with radiotherapy and temozolomide in newly diagnosed high-grade gliomas (ClinicalTrials gov, 2023). These are not silica-phenolic trials, but they are clinically informative because they show where the translational bottlenecks move once phenolic chemistry enters human testing: formulation tolerability, route-specific exposure, pharmacokinetics, and realistic efficacy claims.

The translational message is therefore sobering but useful. Silica nanoparticles have a human track record in selected imaging applications, and phenolic nanomedicines have early clinical exposure in selected formulations, but the integrated MSN-phenolic redox oncology concept remains preclinical. This gap reinforces the need for exposure-aware design, stronger toxicity packages, and conservative claims about tumor-selective redox behavior.

## 7 Discussion and outlook

MSN–phenolic systems are best viewed as interfacial technologies: pore architecture and surface chemistry often determine whether phenolics behave as released small molecules, contact-active surfaces, or components of multi-trigger logic. The dual redox model provides a clinically oriented lens: can a platform plausibly achieve tumor-localized sensitization without inducing systemic antioxidant shielding? Answering this requires coupling materials characterization to biodistribution and time-resolved redox phenotyping—not merely demonstrating uptake or cytotoxicity.

Near-term opportunities include positioning polyphenols as redox adjuvants within combination strategies (chemotherapy, photodynamic therapy, or nucleic-acid co-delivery) where success can be quantified as incremental benefit per delivered dose. Yet the field is unlikely to advance by rhetoric alone. It will yield greater translational value from standardizing redox evidence, defining toxicity boundaries, and clarifying when a formulation is intended to sensitize tumors versus protect normal tissue. In that sense, the most important revision prompted by peer review is conceptual discipline: dual redox behavior must be treated as a testable systems claim rather than a convenient narrative label.

Ultimately, exposure-aware design, reproducible manufacturing, clinically relevant safety packages, and mechanism-linked redox metrics are the decisive levers for moving silica-phenolic redox technologies toward clinical relevance. Progress will likely be stepwise: first by establishing robust preclinical evidence that spatially confined redox programming is real, and only then by attempting to translate it into human oncology.
