# Supplementary material for: Silica–phenolic hybrid nanocarriers in redox oncology: interfacial mechanisms and translational barriers
Source: Front Bioeng Biotechnol. 2026 May 4;14:1814492. doi: 10.3389/fbioe.2026.1814492 (PMC13201897; doi:10.3389/fbioe.2026.1814492)
Supplement: Supplementary file 1 [file Table1.docx]

**Supplementary Material**

**Supplementary Table S1 | Minimal reporting checklist for MSN–phenolic systems.**

This checklist summarizes the minimum information that should be reported to improve reproducibility, clarity in peer review, and translational interpretation of mesoporous silica nanoparticle (MSN)–phenolic systems.

| **Category** | **Minimal items to report** | **Why it matters** |
| --- | --- | --- |
| **Platform definition** | Silica type; intended route; design rationale | Enables comparability and risk assessment |
| **Particle morphology** | TEM/SEM imaging with quantitative size and morphology statistics | Core size and morphology influence cellular uptake, biodistribution, and clearance |
| **Colloidal behavior** | DLS size, polydispersity index (PDI), and stability in relevant media | Predicts aggregation, sedimentation, and protein-corona effects under biological conditions |
| **Surface charge** | Zeta potential with buffer composition and pH explicitly reported | Supports interpretation of colloidal stability, targeting behavior, and corona formation |
| **Porosity metrics** | BET surface area, pore volume, and pore-size distribution | Links pore architecture to phenolic loading, confinement, and release kinetics |
| **Surface chemistry** | FTIR/XPS or equivalent evidence for functional groups, ligands, or gating components | Confirms that the intended interfacial design was actually obtained |
| **Organic fraction** | TGA, elemental analysis, or equivalent quantification of grafting density/organic content | Quantifies the functional layer, payload interface, and batch comparability |
| **Template residuals** | Evidence of surfactant/template removal and residual-contaminant control | Residual surfactants or solvents can confound toxicity and redox readouts |
| **Payload identity** | Phenolic identity, purity, degradation status, or extract standardization | Avoids black-box interpretation of phenolic activity |
| **Loading metrics** | Loading wt%, encapsulation/loading efficiency, and batch-to-batch variability | Supports reproducibility, dose interpretation, and scale-up assessment |
| **Release design** | Release profiles under physiological and tumor-relevant pH, sink conditions, and trigger-response curves when gating is claimed | Provides interpretable kinetics and tests whether release is biologically relevant |
| **Redox evidence** | At least two orthogonal redox readouts with time courses (e.g., ROS kinetics plus GSH/GSSG or lipid peroxidation) | Distinguishes antioxidant buffering, pro-oxidant sensitization, and signaling modulation |
| **Controls** | Free phenolic, blank MSN, functionalized no-payload MSN, and relevant vehicle/media controls | Enables causal attribution to cargo, carrier, and interface |
| **In vivo readiness** | Dose, route, schedule, pharmacokinetic/biodistribution metrics, and tumor/non-tumor exposure, where applicable | Prevents over-claiming targeting and supports exposure-aware interpretation |
| **Safety** | Hemocompatibility, basic hematology/biochemistry, organ histology, inflammatory markers, and dose-limiting observations when applicable | Defines tolerability and identifies off-target or organ-level risk |

*Abbreviations: BET, Brunauer–Emmett–Teller; DLS, dynamic light scattering; EA, elemental analysis; FTIR, Fourier-transform infrared spectroscopy; GSH/GSSG, reduced/oxidized glutathione; MSN, mesoporous silica nanoparticle; PDI, polydispersity index; SEM, scanning electron microscopy; TEM, transmission electron microscopy; TGA, thermogravimetric analysis; XPS, X-ray photoelectron spectroscopy.*
